# Patch-Based Far-Infrared Radiation (FIR) Therapy Does Not Impact Cell Tracking or Motility of Human Melanoma Cells In Vitro

**DOI:** 10.3390/cimb46090599

**Published:** 2024-09-11

**Authors:** Francesca Pacifici, Francesca Chiereghin, Michele D’Orazio, Gina Malatesta, Marco Infante, Federica Fazio, Chiara Bertinato, Giulia Donadel, Eugenio Martinelli, Antonino De Lorenzo, David Della-Morte, Donatella Pastore

**Affiliations:** 1 Department of Human Sciences and Promotion of the Quality of Life, San Raffaele Roma Open University, 00166 Rome, Italy; francesca.pacifici@uniroma2.it (F.P.); francescachiereghin93@gmail.com (F.C.); david.dellamorte@uniroma2.it (D.D.-M.); 2 Interdisciplinary Center for Advanced Studies on Lab-on-Chip and Organ-on-Chip Applications (IC-LOC), University of Rome Tor Vergata, 00133 Rome, Italy; michele.d.orazio@uniroma2.it (M.D.); martinelli@ing.uniroma2.it (E.M.); 3 Department of Electronic Engineering, University of Rome Tor Vergata, 00133 Rome, Italy; 4 Department of Biomedicine and Prevention, University of Rome Tor Vergata, 00133 Rome, Italy; g.malatesta@outlook.it (G.M.); delorenzo@uniroma2.it (A.D.L.); 5 Section of Diabetes & Metabolic Disorders, UniCamillus, Saint Camillus International University of Health Sciences, Via di Sant’Alessandro 8, 00131 Rome, Italy; marco.infante@unicamillus.org; 6 Department of Medical and Surgery Sciences, University “Magna Graecia” of Catanzaro, 8810 Catanzaro, Italy; federica.fazio@tiscali.it; 7 Department of Cellular, Computational and Integrative Biology-CIBO, University of Trento, 38123 Trento, Italy; chiara.bertinato@studenti.unitn.it; 8 Department of Clinical Sciences and Translational Medicine, University of Rome Tor Vergata, 00133 Rome, Italy; donadel@uniroma2.it; 9 Department of Neurology, Evelyn F. McKnight Brain Institute, Miller School of Medicine, University of Miami, Miami, FL 33136, USA

**Keywords:** Far-Infrared Radiation, FIR, patch-based FIR therapy, MAPKs, cell migration, wound healing assay, M14/C2 cell line

## Abstract

Far-Infrared Radiation (FIR) is emerging as a novel non-invasive tool for mitigating inflammation and oxidative stress, offering potential benefits for certain medical conditions such as cardiovascular disease and chronic inflammatory disorders. We previously demonstrated that the application of patch-based FIR therapy on human umbilical vein endothelial cells (HUVECs) reduced the expression of inflammatory biomarkers and the levels of reactive oxygen species (ROS). Several in vitro studies have shown the inhibitory effects of FIR therapy on cell growth in different cancer cells (including murine melanoma cells), mainly using the wound healing assay, without direct cell motility or tracking analysis. The main objective of the present study was to conduct an in-depth analysis of single-cell motility and tracking during the wound healing assay, using an innovative high-throughput technique in the human melanoma cell line M14/C2. This technique evaluates various motility descriptors, such as average velocity, average curvature, average turning angle, and diffusion coefficient. Our results demonstrated that patch-based FIR therapy did not impact cell proliferation and viability or the activation of mitogen-activated protein kinases (MAPKs) in the human melanoma cell line M14/C2. Moreover, no significant differences in cell motility and tracking were observed between control cells and patch-treated cells. Altogether, these findings confirm the beneficial effects of the in vitro application of patch-based FIR therapy in human melanoma cell lines, although such effects need to be confirmed in future in vivo studies.

## 1. Introduction

The human body is continuously exposed to electromagnetic radiation, with infrared radiation being widely used in healthcare. According to the International Commission on Illumination (CIE), infrared radiation (IR) can be divided into three subgroups: Near-Infrared Radiation (NIR), Mid-Infrared Radiation (MIR), and Far-Infrared Radiation (FIR) [1,2]. Among these, FIR transmits energy that can be perceived by skin thermoreceptors as radiant heat, promoting both thermal and non-thermal health benefits. The biological effect of FIR energy transmission is promoting the vibration and rotation of molecular bonds, leading to an increase in epidermal temperature without significant skin heating [1]. Several human studies have reported the benefits of FIR therapy. In particular, FIR applied through FIR-based saunas has been shown to exert beneficial effects on cardiovascular health by reducing oxidative stress in patients with chronic heart failure [3]. Moreover, FIR sauna treatments have been shown to improve physical health in patients with type 2 diabetes mellitus by alleviating clinical stress and fatigue [4]. FIR saunas have also been demonstrated to reduce pain, stiffness, and fatigue in patients with both rheumatoid arthritis and ankylosing spondylitis [5]. In addition to saunas, FIR devices are widely used to treat allergic rhinitis and exercise-induced muscle damage in athletes [6,7].

Since FIR is also emitted by our body, advances in technology have led to the creation of coverings (clothes, patches) integrated with small particles of FIR-emitting ceramics. These ceramics absorb the FIR emitted by our body and then re-emit it back to the skin, promoting both the thermal and non-thermal effects of FIR [1]. Herr et al. demonstrated that the application of bio-ceramic wraps in patients with lower limb venous ulcers significantly improves ulcer healing [8]. Recently, van Kraaij et al. reported the benefits and tolerability of FIR patches on both skin microcirculation and local oxygen consumption in healthy subjects [9].

Numerous in vitro studies have shown the beneficial effects of FIR using different devices. FIR heat lamps affect microcirculation by stimulating endothelial nitric oxide synthase (eNOS) in human umbilical vein endothelial cells (HUVECs) [10]. Moreover, we reported that FIR patch therapy reduced inflammation and oxidative stress in HUVEC and kidney (HEK293) cell lines exposed to tumor necrosis factor (TNF)-α [11]. Akasaki et al. demonstrated that a dry FIR sauna increased eNOS levels and improved angiogenesis in mice with hindlimb ischemia [12]. Furthermore, Toyokawa et al. showed that FIR irradiation on the wounded skin in a mouse model accelerated wound healing, mediated by the increased expression of transforming growth factor-beta1 (TGF-beta1) at the lesion site [13].

Despite the beneficial effects of FIR therapy, we questioned whether FIR patch application might impact the molecular processes related to cell migration and motility. Although FIR therapy improves wound healing in normal tissue, it can also inhibit the proliferation and/or migration of several cell cancer lines, such as human breast cancer cells [14], human tongue squamous carcinoma cells (HSC3), human gingival squamous carcinoma cells (Sa3), human lung carcinoma cells (A549) [1,15], and murine melanoma cells [16], by acting on different signaling pathways. However, there is limited evidence regarding the in-depth analysis of FIR’s impact on cell tracking and motility, two relevant parameters driving the malignancy of cancer cells. To further confirm the safety of FIR patch application, the aim of the present in vitro study was to analyze the impact of patch-based FIR therapy on single-cell tracking and motility in human melanoma cells. To achieve this goal, we employed an innovative high-throughput experimentation to obtain meaningful information on the behavior of living cells. By using advanced computer algorithms, we detected cell trajectories and automatically quantified different cell motility descriptors. Moreover, we evaluated the impact of patch-based FIR therapy on the activation of mitogen-activated protein kinases such as p38, which has been shown to promote migration and metastatic activity in BRAF (V600E) mutant melanoma cells [17].

## 2. Materials and Methods

### 2.1. Cell Culture and Patch-Based FIR Therapy

The human melanoma cell line M14/C2 clone [18] was kindly provided by Dr. Grazia Graziani from the University of Rome Tor Vergata. Cells were cultured in RPMI 1640 (Thermo Fisher Scientific, Waltham, MA, USA), supplemented with 10% heat-inactivated fetal bovine serum (HI FBS) (Corning, NY, USA) and 100 U/mL penicillin/streptomycin (Thermo Scientific, Waltham, MA, USA).

FIR patches were kindly provided by D. FENSTEC s.r.l. (Altavilla Vicentina, VI, 36077, Italy). The patches were applied to the external side of the cell culture plates for the time indicated in the different experimental procedures. Polystyrene did not interact with FIR emission. FIR patches did not release any pharmacologic substances nor did they produce thermal shock.

### 2.2. Scratch Assay

**Figure 1:**
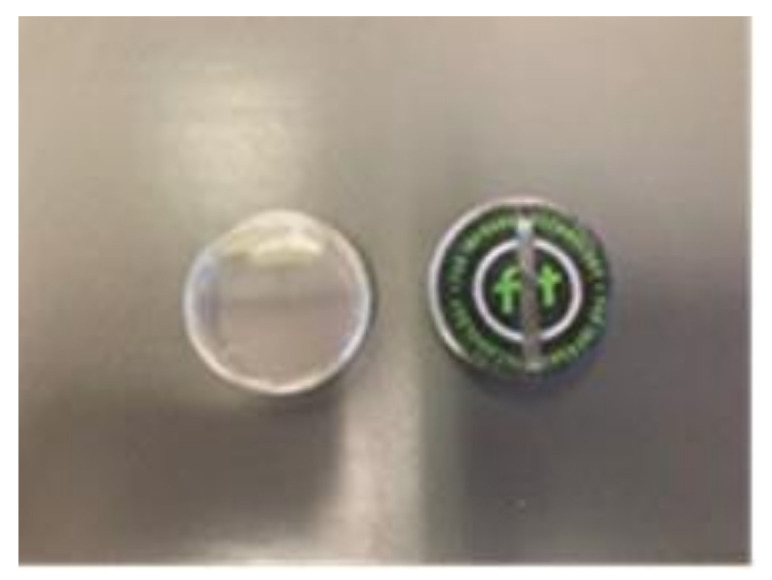
Cell dishes with and/or without FIR patch. Dish without (**left**) and with (**right**) the application of the FIR patch.

To study cell migration, a scratch assay was performed. M14/C2 cells (1.3 × 10^6^) were plated in 35 mm culture dishes with or without FIR patches adapted for the assay, as shown in Figure 1. After 48 h, the culture medium was removed, and cells were carefully scratched by using a plastic 200 µL pipette tip to draw a straight line in the cell monolayer. Cells were then washed twice with Phosphate-Buffered Saline (PBS, Sigma Aldrich, Saint Louis, MO, USA), and fresh medium was added. Cells were observed with time-lapse microscopy for 72 h at a temperature of 37 °C and in humidified air containing 5% CO_2_.

### 2.3. Cell Proliferation Assay

Human melanoma cell line M14/C2 was cultured and seeded in a 24-well plate (60.000 cells/well) with or without FIR patches. After 24 and 48 h, the cells were washed with PBS and detached from the culture plates using PBS/EDTA (200 mg/L). Subsequently, the cells were counted using trypan blue (Sigma Aldrich, Saint Louis, MO, USA) and a Neubauer counting chamber.

### 2.4. Cell Viability Assay

To assess cell viability, we used the MTT (3-(4,5-dimethylthiazol-2-yl)-2,5-diphenyltetrazolium bromide) assay (Sigma Aldrich, Saint Louis, MO, USA), as previously reported [19]. Briefly, 1 × 10^4^ cells/well were seeded in a 96-well plate for 24 and 48 h with or without FIR patches. The culture medium was then removed, and 5 mg/mL of MTT was added and incubated for 3 h at 37 °C. After the incubation period, the formazan crystals formed were dissolved in DMSO (dimethyl sulfoxide) (Sigma Aldrich, Saint Louis, MO, USA), and formazan content was assessed by measuring the absorbance at 570 nm.

### 2.5. Platform for the Analysis of Cell Motility

**Figure 2:**
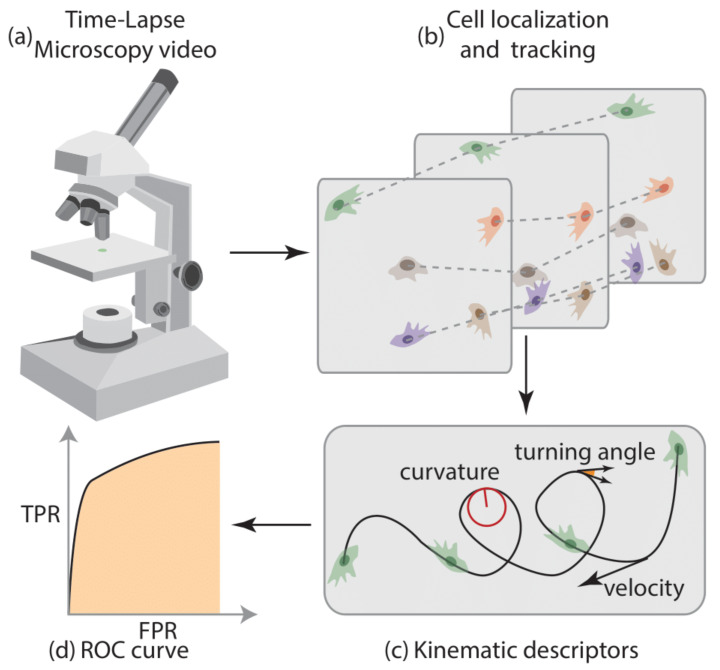
Platform for cell motility analysis. Platform for cell motility analysis, comprising time-lapse microscopy video acquisition (**a**), cell localization and tracking (**b**), kinematic descriptor extraction (**c**), and ROC curve analysis (**d**).

The entire platform for the analysis of cell motility is shown in Figure 2. The process begins with the customized acquisition of time-lapse microscopy videos (Figure 2a). Cells are tracked throughout the videos (Figure 2b), and cell motility descriptors are extracted (Figure 2c). Finally, the separability of the two classes was quantified using the area under the receiver operating characteristic (ROC) curve of the cell kinematic descriptors (Figure 2d). 

### 2.6. Time-Lapse Microscopy Details

A total of 6 videos were acquired with a custom small-scale inverted microscope, whose details are described elsewhere [20,21,22]. Ad hoc software was developed in Matlab 2017^®^ (MathWorks^®^, Natick, MA, USA) to obtain full control over acquisition settings. Each video was acquired for 72 h at 1 frame per minute and a theoretical spatial resolution of 0.66 µm/px. We acquired three videos for each experimental condition (i.e., control and FIR patch-treated).

### 2.7. Cell Localization and Tracking

Cell localization and tracking were performed using Cell-Hunter, validated Matlab^®^ software (https://www.mathworks.com/products/matlab.html, accessed on 1 June 2023) [23,24]. Cells were located using the circle Hough transform (CHT) [25], with a radius range between 5 and 8 µm. For each pair of positions in two consecutive frames, the distance was computed, and the Kuhn–Munkres algorithm [26] was used to find a suboptimal solution, minimizing the overall cost. The final output from Cell-Hunter software [27,28] consisted of a set of cell trajectories.

### 2.8. Cell Motility Analysis

Cell tracks lasting less than 50 min were discarded to ensure the statistical significance of the extracted features. A smoothing spline approximation was performed to mitigate errors linked to pixel quantization and localization. The new set of positions (*x_s_*, *y_s_*) was used to compute the following descriptors for their already proven significance:

a. Tangential speed norm

(1) $$v\left(t_{k}\right)=\sqrt{{\left(\frac{x\left(t_{k+1}\right)-x\left(t_{k}\right)}{\left(t_{k+1}-t_{k}\right)}\right)}^{2}+{\left(\frac{y\left(t_{k+1}\right)-y\left(t_{k}\right)}{\left(t_{k+1}-t_{k}\right)}\right)}^{2}}$$

b. Curvature

(2) $$\chi \;\left(t_{k}\right)=\frac{\left|x_{s}^{\prime }y_{s}^{\prime \prime }-y_{s}^{\prime }x_{s}^{\prime \prime }\right|}{{\left[{\left(x_{s}^{\prime }\right)}^{2}+{\left(y_{s}^{\prime }\right)}^{2}\right]}^{\frac{3}{2}}}$$

c. Turning angle

(3) $$\vartheta \left(t_{k}\right)=\tan^{-1}\left(\frac{v_{x}}{v_{y}}\right)$$

d. Diffusion coefficient

(4) $$D=\frac{1}{4}\;e^{y_{0}}$$

Notably, *y_0_* represents the intercept on the *y*-axis of the linear FIR in the logarithm space of the mean square displacement, as described by Sbalzarini et al. [29]. The mean was extracted from tangential speed norm, curvature, and turning angle signals to obtain global descriptors of the cell trajectories.

### 2.9. Western Blot Analysis

Human melanoma cells (M14/C2 clone) were plated and treated as reported for the scratch assay. After 72 h, they were collected and processed for Western blot analysis according to Pacifici et al. [19]. Briefly, protein extraction was conducted using cold lysis buffer containing 20 mM Tris (pH 7.6), 137 mM NaCl, 1.5% NP40, 1 mM MgCl_2_, 1 mM CaCl_2_, 10% glycerol, 2 mM phenylmethylsulfonyl fluoride (PMSF), phosphatase inhibitor cocktails (Sigma Aldrich, Saint Louis, MO, USA), and protease inhibitor cocktail tablets (Roche Diagnostics GmbH, Mannheim, Germany). All samples were kept on ice for 30 min and then centrifuged at 14,000 rpm for 30 min. The supernatants were collected and quantified by the Bradford protein assay according to the manufacturer’s protocol. Then, 50 μg of proteins was loaded onto a 4–12% precast gel (Thermo Scientific, Waltham, MA, USA) and transferred onto nitrocellulose membrane using a Trans-Blot Turbo Transfer System (Bio-Rad Laboratories, Milan Italy). Membranes were blocked at room temperature with 5% non-fat dry milk and probed with the following primary antibodies: p-p38 (9216S), p38 (9212), pERK1/2 (4370S), total ERK1/2 (137F5) (1:1000 Cell Signaling Technologies, Denver, MA, USA), and vinculin (sc73614, 1:200, Santa Cruz Biotechnology, Santa Cruz, CA, USA). The antibody complexes were acquired and quantified using a Gel DocTM XR + with Image LabTM Software 6.1 (Bio-Rad Laboratories, Milan, Italy).

### 2.10. Statistical Analysis

All data were analyzed by using GraphPad Prism 10.0 (La Jolla, CA, USA). For cell proliferation and cell viability, a two-way ANOVA with mixed-effect analysis and multiple comparisons was used. The statistical analysis and significance for the Western blot analysis were evaluated using an unpaired two-tailed Student’s *t*-test. All data are expressed as mean ± SEM, as indicated. *p*-values < 0.05 were considered statistically significant.

## 3. Results

### 3.1. Effects of Patch-Based FIR Therapy on Cell Proliferation and Cell Survival

**Figure 3:**
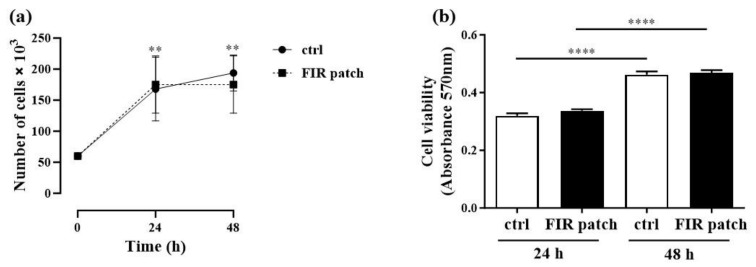
Cell proliferation and survival assay: (**a**) 60,000 cells/well were plated in a 24-well plate for the proliferation assay. After 24 h and 48 h, cells were detached and counted. (**b**) A total of 1 × 10^4^ cells/well was seeded in a 96-well plate for 24 h and 48 h for the cell survival assay (MTT assay), as reported in Section 2. Data are expressed as mean ± SEM (standard error of the mean). ** *p* < 0.005 (vs. time 0); **** *p* < 0.0001 (vs. time 0) (*n* = 4). All *p*-values were obtained by two-way ANOVA. Abbreviations: ctrl, control cells; patch, FIR patch-treated cells.

To analyze the potential effects of FIR on human melanoma cell growth, we conducted a proliferation assay as described in the Section 2. As shown in Figure 3a, a significant increase in cell proliferation was observed at 24 h (*p* < 0.005) and at 48 h (*p* < 0.0001) compared to that at the baseline (time 0) in both control (ctrl) and FIR patch-treated cells. As expected, no significant differences were observed between the two study groups. We then moved forward to analyze the impact of FIR on cell viability at 24 and 48 h using the MTT assay. Consistent with the proliferation assay, we observed a significant increase in cell viability at 48 h compared to the 24 h time point in both control (ctrl) and FIR patch-treated cells (*p* < 0.0001) (Figure 3b). However, no significant differences were found between the two study groups. These data suggest that patch-based FIR therapy did not impact cell proliferation, even in a cell subtype with high replicative potential.

### 3.2. Cell Tracking and Motility Assessment

**Figure 4:**
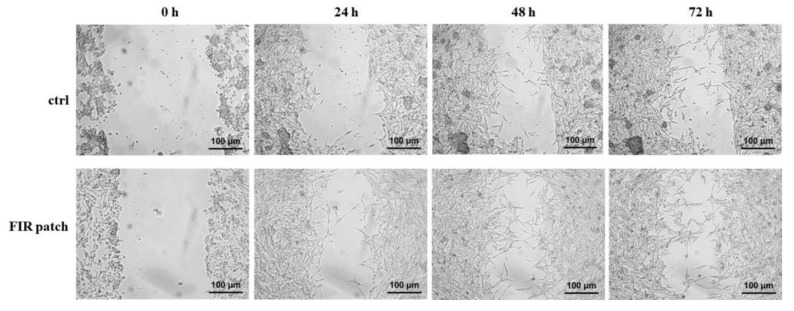
Scratch assay. Cells (1.3 × 10^6^) were plated in 35 mm culture dishes for 48 h. Then, a scratch was performed, and cells were analyzed until 72 h. Abbreviations: ctrl, control cells; patch, FIR patch-treated cells (*n* = 3).

To assess cell migration, a scratch assay was performed according to the protocol described in the Section 2. As shown in Figure 4, we did not detect significant differences in cell migration between the control (ctrl) and FIR patch-treated cells during the assay. A magnified version of the images in Figure 4 is presented in Supplementary Figure S1, allowing for detailed cell visualization. These findings suggest that patch-based FIR therapy did not exert any effect on human melanoma cell (M14/C2) migration.

**Figure 5:**
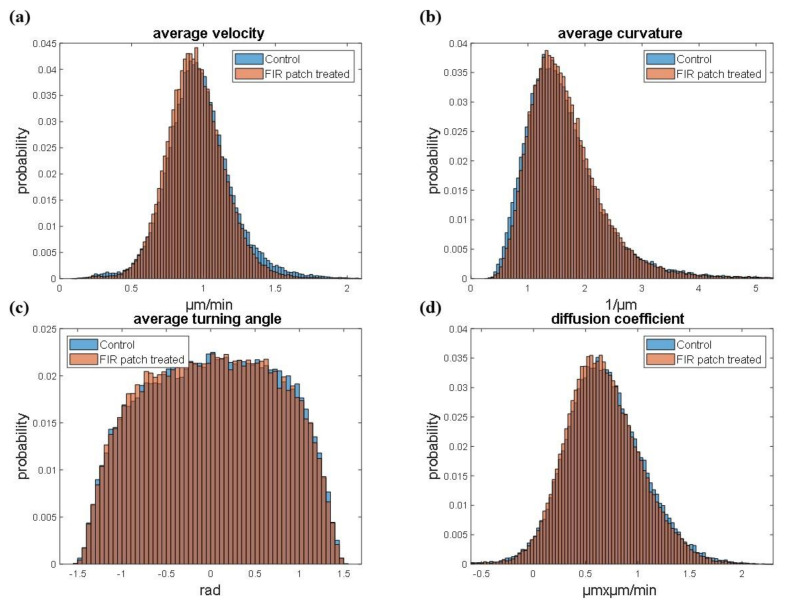
Motility descriptors. Normalized histograms of average velocity (**a**), average curvature (**b**), average turning angle (**c**), and diffusion coefficient (**d**) under the two conditions (absence of exposure to FIR patch and exposure to FIR patch). The histogram represents the merge between control cells and FIR patch-treated cells (*n* = 3).

To further validate these findings, we compared the two cell groups (control cells in blue and FIR patch-treated cells in orange) using four different motility descriptors. Specifically, Figure 5a shows the average single-cell velocity, Figure 5b reports the average curvature, Figure 5c describes the average turning angle, and Figure 5d displays the diffusion coefficient. The proposed methodology involves a single-cell kinematic analysis of three different videos under both conditions, enabling a robust statistical analysis of the results. As shown, the distributions of the features under the two conditions (absence of exposure to FIR patch and exposure to FIR patch) are almost completely overlapped, further confirming that FIR patch application did not affect cell motility, as previously observed in the scratch assay.

**Figure 6:**
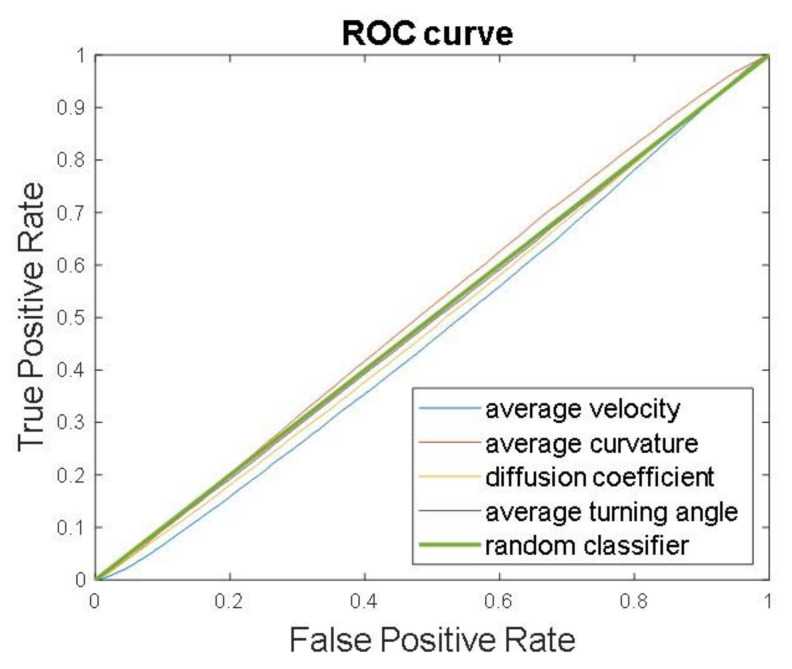
Receiver operating characteristic curve (ROC curve) of cell motility descriptors (i.e., average velocity, average curvature, average turning angle, and diffusion coefficient) compared with the ROC curve obtained with a random classifier (*n* = 4).

To further investigate the inseparability of the two classes based on the cell motility features, we computed the receiver operating characteristic curve (ROC curve) for each feature (Figure 6). The ROC curve is a graphical representation that shows the discrimination ability of a binary classifier system as its discrimination threshold is varied. It is created by plotting the true positive rate (sensitivity) against the false positive rate (1-specificity) at various threshold settings. The area under the ROC curve (AUC) provides a single measure of overall classification performance: a value of 0.5 indicates a model with no discrimination ability, equivalent to random guessing, while a value of 1.0 indicates perfect classification. The curves in Figure 6 provide evidence that all the features considered are close to the performance of a random guess classifier. This indicates that the features cannot be used to assess if the test sample was drawn from one of the two populations, i.e., control or FIR-patch-treated cells.

### 3.3. Patch-Based FIR Therapy Does Not Promote the Activation of ERK1/2 MAPK Pathway

**Figure 7:**
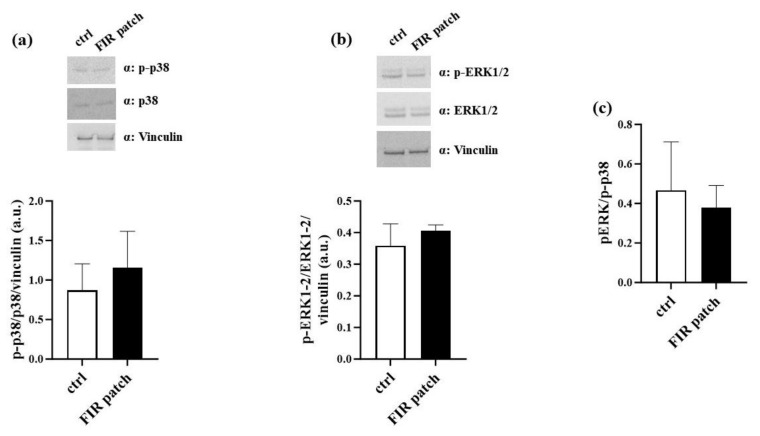
Impact of patch-based FIR therapy on ERK1/2 MAPK pathway (Western blot analysis). Expression levels of MAPK. (**a**) p-p38 and p-38 protein levels; (**b**) p-ERK1/2 and ERK1/2 expression levels; (**c**) p-ERK1/2 and p-p38 ratio. All data were normalized using vinculin as an internal control. Data are expressed as mean ± SEM (*n* = 3). Abbreviations: ctrl, control cells; MAPK, mitogen-activated protein kinase; pERK1/2, phosphorylated extracellular signal-regulated kinase ½ (*n* = 4). All *p*-values were obtained by a two-tailed Student’s *t*-test.

We also analyzed the expression levels of the kinase enzymes involved in promoting cell migration. Specifically, it was demonstrated that phospho-p38 (p-p38) promotes melanoma cell proliferation and migration [30]. Based on our findings, we analyzed the expression levels of p-p38 in control cells and FIR patch-treated cells. In keeping with the abovementioned findings, we did not observe significant differences in the p-p38 levels between control cells and FIR patch-treated cells (Figure 7a). Similarly, we analyzed the activation levels of the ERK1/2 (extracellular signal-regulated kinase 1/2) MAPK (mitogen-activated protein kinase) pathway, which is also involved in cell proliferation and migration [30]. As shown in Figure 7b, we did not observe significant changes in the activation levels of the ERK1/2-MAPK pathway between control cells and FIR patch-treated cells. It was reported that an increased p-ERK/p-p38 ratio may be predictive of melanoma progression in vivo [30]. To further confirm that FIR patch application did not impact the invasiveness or migration of melanoma cell lines, we also evaluated the pERK/p-p38 ratio. As shown in Figure 7c, no significant increase in the ratio was observed. Altogether, these results suggest that patch-based FIR therapy did not impact the migration and proliferation of human melanoma cells M14/C2.

## 4. Discussion

In the present study, we found that patch-based FIR therapy did not impact the viability or proliferation of human melanoma cell lines. Moreover, we found that patch-based FIR therapy did not affect the motility or migration of these cells, as assessed by both a scratch assay and time-lapse microscopy. Specifically, FIR patch application did not increase the average velocity of the treated cells during wound healing. Additionally, we did not observe significant differences in the activation of the p38 and ERK1/2-MAPK pathways between the control cells and FIR patch-treated cells.

FIR has been used as a novel non-invasive therapy for various medical conditions due to its thermal and non-thermal effects [1]. Several studies have reported that FIR patches have been widely and safely used for different pathological conditions. Ricci et al. tested FIR patch therapy in 20 subjects affected by shoulder tendinopathies [31]. The study showed an improvement in painful symptoms and an amelioration in rotator cuff tendinopathies. A retrospective observational study analyzed the effects of FIR patch therapy on musculoskeletal and neurological symptoms [32]. The authors reported that FIR patch application improved pain, physical status, and related symptoms, overall enhancing the quality of life of all enrolled patients, with no adverse events recorded. In a study conducted by Ricci et al., it was demonstrated that the application of FIR patches for 14 days reduced pain levels and improved lifestyle, suggesting that FIR patch therapy can be used as an alternative non-pharmacological treatment for pain relief [33].

Moreover, FIR therapy has been shown to improve vascular endothelial function [34], inhibit vascular endothelial inflammation [35], promote ischemia-induced angiogenesis, and restore high-glucose-suppressed endothelial progenitor cell functions [12,36].

In the present study, we found that patch-based FIR therapy did not affect the migration of the human melanoma cell line. However, we did not observe a reduction in cell proliferation, as has been reported by other studies conducted on cell cultures [14,16]. This discrepancy may be due to the different methods of FIR therapy administration. Several systems have been used for FIR application, ranging from FIR radiant-panel incubators [37] to FIR ceramic powder composed of microsized particles [16]. These techniques are based on direct cell irradiation rather than on the reflection of infrared radiation emitted by human skin [38]. A limitation of the method employed in the present study may be the placement of FIR patches on the upper and lower surfaces of the cell culture dish but not on the other sides. Thus, some infrared radiation may not have been properly reflected. However, this remains the most reliable system for mimicking the commercially available FIR patches in vitro, as infrared radiation is not emitted by the patch itself; rather, the infrared radiation emitted by the cells is exploited for FIR thermal effects.

To the best of our knowledge, this is the first study reporting the use of a high-throughput technique for analyzing the effect of FIR therapy on cell migration. Indeed, in addition to the scratch assay, we conducted an in-depth time-lapse microscopy analysis. This innovative approach demonstrated that various cell motility descriptors, such as average velocity, average curvature, average turning angle, and diffusion coefficient, showed no significant difference when comparing control cells and FIR patch-treated cells. This finding suggests that patch-based FIR therapy did not impact the migration of human melanoma cells, further confirming the results of the scratch assay.

Cell migration is regulated by different factors, including the mitogen-activated protein kinases p38 and ERK1/2 [39]. The activation of these kinases has been associated with the invasive behavior of the human melanoma cell line M14 [40]. In our experimental study, we investigated the involvement of p38 and pERK1/2 in M14/C2 cell migration. As reported, FIR patches did not significantly increase the activation of either p38 or pERK1/2, although they did reduce the pERK/p-p38 ratio. This finding suggests that patch-based FIR therapy did not promote invasive behavior in the M14/C2 cell line.

Based on our previous results obtained on HUVECs, we speculate that FIR patch application may reduce oxidative stress and inflammation, thereby improving the cellular environment and leading to reductions in proliferation and migration. Moreover, based on the scientific literature, FIR administration might inhibit Heat Shock Protein (Hsp) 70, which is increased in melanoma tumors and exhibits tumor-selective cytotoxicity [41] by reducing migration, invasion, and metastasis. However, further studies are needed to validate our results and to better clarify the molecular mechanisms underlying the migration of human melanoma cells.

The main limitation of the present study is that it investigated the effects of patch-based FIR therapy only on human melanoma cell lines in vitro.

The novelty of the present study lies not only in the FIR administration method but also in the advanced analysis performed to evaluate cell tracking and migration (which is considered one of the least-invasive techniques) and in the high-throughput experimentation employed to obtain meaningful information on the behavior of living cells. Moreover, the use of advanced computer algorithms allowed us to detect cell trajectories and automatically quantify different cell motility descriptors.

Finally, our findings confirm the beneficial and safe effects of the in vitro application of FIR patches in human melanoma cell lines, even though these effects need to be confirmed in future in vivo studies.

## Data Availability

Data used and analyzed in the current study will be made available by the corresponding author (D.P.) upon reasonable request.

## Supplementary Materials

The following supporting information can be downloaded at: https://www.mdpi.com/article/10.3390/cimb46090599/s1. Supplementary Figure S1: Magnified version of scratch assay.
