# Supplementary material for: Patch-Based Far-Infrared Radiation (FIR) Therapy Does Not Impact Cell Tracking or Motility of Human Melanoma Cells In Vitro
Source: Curr Issues Mol Biol. 2024 Sep 11;46(9):10026–37. doi: 10.3390/cimb46090599 (PMC11429816; doi:10.3390/cimb46090599)
Supplement: Supplementary file 1 [file cimb-46-00599-s001.zip › cimb-3169305-supplementary.pdf]

Supplementary Figure 1

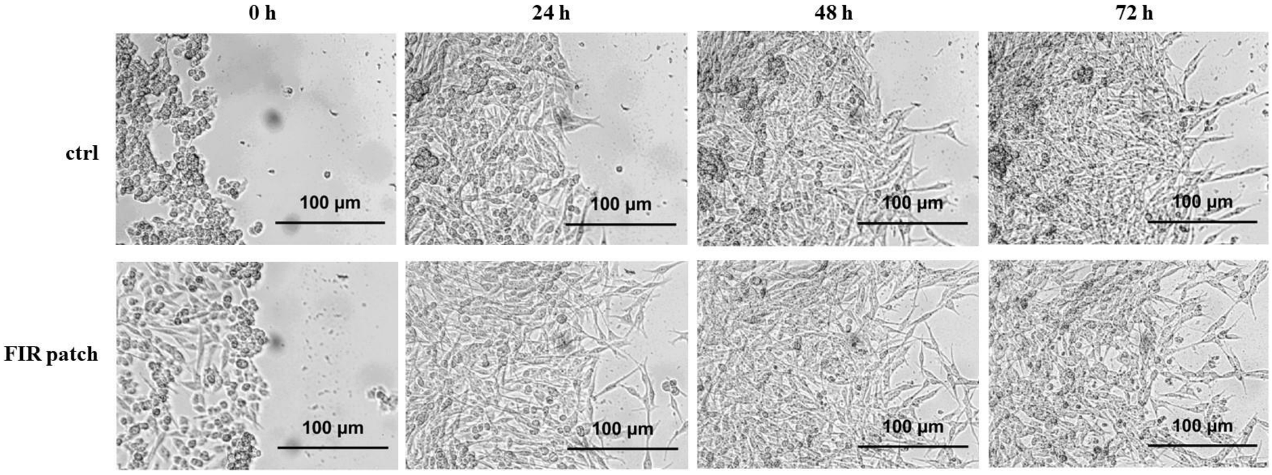

Figure S1: Time-lapse image magnification for better cell visualization. These images are obtained by cropping the top-left quarter of the corresponding images in Figure 4, allowing a better visualization of cell details.
